# Involvement of BcElp4 in vegetative development, various environmental stress response and virulence of *Botrytis cinerea*

**DOI:** 10.1111/1751-7915.12720

**Published:** 2017-05-05

**Authors:** Wenyong Shao, Chiyuan Lv, Yu Zhang, Jin Wang, Changjun Chen

**Affiliations:** ^1^ College of Plant ProtectionNanjing Agricultural UniversityNanjing210095China

## Abstract

The *Saccharomyces cerevisiae* Elongator complex consisting of the six Elp1‐Elp6 proteins has been proposed to participate in three distinct cellular processes: transcriptional elongation, polarized exocytosis and formation of modified wobble uridines in tRNA. In this study, we investigated the function of BcElp4 in *Botrytis cinerea*, which is homologous to *S. cerevisiae Elp4*. A *bcelp4* deletion mutant was significantly impaired in vegetative growth, sclerotia formation and melanin biosynthesis. This mutant exhibited decreased sensitivity to osmotic and oxidative stresses as well as cell way‐damaging agent. Pathogenicity assays revealed that BcElp4 is involved in the virulence of *B. cinerea*. In addition, the deletion of *bcelp4* led to increased aerial mycelia development. All these defects were restored by genetic complementation of the *bcelp4* deletion mutant with the wild‐type *bcelp4* gene. The results of this study indicated that BcElp4 is involved in regulation of vegetative development, various environmental stress response and virulence in *B. cinerea*.

## Introduction

The transcriptional elongation complex was first identified based on its direct association with the RNA polymerase II holoenzyme in yeast (Otero *et al*., 1999). The protein complex is composed of two sub‐complexes: one made of the Elp1, Elp2 and Elp3 subunits and an other one constituted by Elp4, Elp5 and Elp6 subunits forming the hexameric holo‐Elongator (Petrakis *et al*., 2004). Elp3 forms a stable complex with the five other polypeptides called Elp1, Elp2, Elp4, Elp5 and Elp6 (Krogan and Greenblatt, 2001) and contains two domains. Previous studies showed that Elp3 contains an iron–sulphur cluster that can bind S‐adenosylmethionine (Paraskevopoulou *et al*., 2006) and may be involved in DNA demethylation (Okada *et al*., 2010). Amyotrophic lateral sclerosis was shown recently to be linked to allelic variants of ELP3 (Simpson *et al*., 2009). Based on sequence comparisons, it was suggested that Elp4 and Elp6 could be inactive orthologues of ancestral ATPases involved in chromatin remodelling (Ponting, 2002). In *Saccharomyces cerevisiae*, deletions of the individual genes that encode the Elongator subcomplex Elp4–6 revealed that only the ELP5 gene is essential for growth (Krogan and Greenblatt, 2001). However, the mammalian ELP4 gene was recently implicated in rolandic epilepsy (Strug *et al*., 2009) and the eye anomaly aniridia (Crolla and van Heyningen, 2002; Kleinjan *et al*., 2002; Zhang *et al*., 2011). In yeast, the Elongator complex functions as a histone acetyltransferase (HAT) complex that was related to the hyper‐phosphorylated elongating form of RNA polymerase II (Glatt *et al*., 2012). The known biological roles of Elongator are in RNAPII‐mediated transcription through the acetylation of histone H3 and H4 in chromatin, the modification of certain tRNAs and the acetylation of alpha‐tubulin (Otero *et al*., 1999; Fellows *et al*., 2000; Jablonowski *et al*., 2001). Previous studies have shown that Elongator was involved in various cellular functions including tRNA modification, transcriptional silencing, sensitivity to DNA damaging agents and histone acetylation (Wittschieben *et al*., 1999; Huang *et al*., 2005; Esberg *et al*., 2006; Li *et al*., 2009; Okada *et al*., 2010). In addition, in yeast, Elongator mutants defective in vegetative growth exhibited sensitivity to rapamycin, high temperature, hydroxyurea, caffeine and various other stresses and were resistant to zymocin (Frohloff *et al*., 2001; Li *et al*., 2009), a protein toxin secreted by the yeast *Kluyveromyces lactis* that kills other yeasts including *S. cerevisiae* (Jablonowski and Schaffrath, 2007). The Elongator was also involved in replication‐coupled nucleosome assembly, transcriptional silencing and polarized secretion (Rahl *et al*., 2005; Li *et al*., 2009).

Elongator complex is conserved in eukaryotes and has also been purified from humans (Hawkes *et al*., 2002). Mutations in the human homologue of yeast *ELP1, IKBKAP/hELP1*, have been shown to cause *Familial Dysautonomia* (FD), a genetic disorder primarily affecting the sensory and autonomic nerve systems (Anderson *et al*., 2001; Slaugenhaupt *et al*., 2001; Gold‐von Simson and Axelrod, 2006). Human IKAP/hELP1 protein is one of the homologues of yeast Elongator proteins (Hawkes *et al*., 2002). Previous reports indicated that mutations of Elongator subunits (ELO1/ELP4, ABO1/ELO2/ELP1 and ELO3/ELP3) in *Arabidopsis* have pleiotropic effects on plant development and growth (Nelissen *et al*., 2005; Chen *et al*., 2006).

*Botrytis cinerea* is a necrotrophic plant pathogenic fungi causing pre‐ and post‐harvest grey mould in multiple plant species, resulting in serious economic losses in fruit, vegetable and ornamental flower production (Elad *et al*., 2004; Willamson *et al*., 2007; An *et al*., 2016). All plant organs, including leaves, flowers, soil storage organs and shoots, are susceptible to *B. cinerea* attacks (Veloukas and Karaoglanidis, 2012). Under a wide range of environmental conditions, *B. cinerea* employs various modes to attack its different hosts (Qin *et al*., 2010). In addition, it can survive as conidia and mycelia or for extended periods of time as sclerotia in plant debris (Willamson *et al*., 2007), resulting in that the control of grey mould is full of challenges. Thus, research on the mechanism of development and pathogenesis of *B. cinerea* could provide strategies to reduce the damage caused by the grey mould. A whole‐genome search uncovered that *B. cinerea* has an orthologue of Elp4 (hereafter named *bcelp4*). Based on the analysis of function of *Elp4* in yeast, we hypothesized that in B.c., *bcelp4* might have significant effects on development and virulence in *B. cinerea*. To clarify this hypothesis, the major target of this study was to explore the biological and biochemical function of *bcelp4* using a target gene deletion strategy. Our study indicated that *bcelp4* plays important roles in regulating the vegetative growth, asexual development, melanin synthesis, various stresses sensitivity and virulence in *B. cinerea*.

## Results

### Characterization of the *bcelp4* gene

The *bcelp4* (BC1G_03169) was originally identified via a homology search of the *B. cinerea* genome database using BLAST with the *elp4* gene from *S. cerevisiae* as a query (Glatt *et al*., 2012). To analyse the size and existence of introns, RNA was extracted from mycelia of the wild‐type strain B05.10 with Tian‐gen Reagent kit (Tian‐gen Biotech. Co., Beijing, China) and was used for reverse transcription with a cDNA synthesis kit (TaKaRa Biotech. Co., Dalian, China). The primer pair P1/P2 (Table S1) generated 1209‐bp and 1297‐bp fragments from cDNA and genomic DNA. Sequence results indicated that the coding region of *bcelp4* has one intron of 88 bp, which was located after the first 451 nucleotides (Fig. S1).

### Generation of *bcelp4* deletion and complemented mutant

To research the biological function(s) of BcElp4 in *B. cinerea*, we constructed a mutant using a homologous recombination strategy (Fig. S2). The deletion mutant was identified from 189 hygromycin‐resistant transformants by PCR and verified with different primer pairs. The P11/P12 primers amplified a 345‐bp fragment from the wild‐type strain B05.10 but did not amplify any fragment from the *bcelp4* deletion mutant. The P13/P14, P15/P16 and P17/P18 primers amplified 981‐bp, 2688‐bp and 2399‐bp fragments from the *bcelp4* deletion mutant, respectively, but did not amplify any fragment from the wild‐type strain B05.10. (Fig. S3). In a southern hybridization assay, when probed with the 706‐bp 3′‐flanking region of *bcelp4*, the *bcelp4* deletion mutant *Δbcelp4* generated a 4243‐bp band, but lacked a 3776‐bp band which was present in the wild‐type strain B05.10.

These results confirm that *Δbcelp4* resulted from the anticipated homologous recombination events at the *bcelp4* locus. The wild‐type *bcelp4* was ectopically integrated into the genome of the complemented strain (*Δbcelp4C*).

### Involvement of *bcelp4* in vegetative growth and sclerotial formation

**Figure 1:**
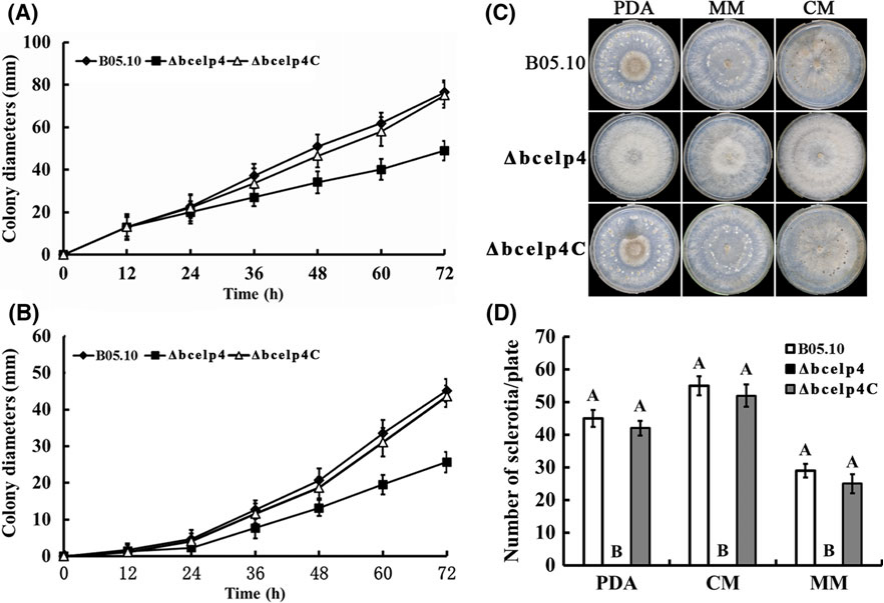
Effect of *bcelp4* deletion on mycelia growth and sclerotial formation. A. Mycelial growth rate of the wild‐type strain B05.10, the *bcelp4* deletion mutant *ΔbcElp4* and the complemented strain *Δbcelp4C* on potato dextrose agar (PDA). Bars denote standard errors from three repeated experiments. B. Mycelial growth rate of each strains on minimal medium (MM). Bars denote standard errors from three repeated experiments. C. Comparison of sclerotial formation among B05.10, *Δbcelp4* and *ΔbcElp4C* after 4 weeks of incubation on PDA, minimal medium (MM) and complete medium (CM) at 25°C in darkness. D. The number of sclerotia was measured on PDA, MM and CM. Bars denote standard deviation from three experiments. Values on the bars followed by the same letter are not significantly different at *P* = 0.05.

To analyse the role of *bcelp4* in vegetative colony growth, each strain was inoculated on potato dextrose agar (PDA), minimal medium (MM) and complete medium (CM) respectively. The growth rate of *Δbcelp4* showed a significant reduction compared with that of the wild‐type strain, but the growth rate did not differ between the complemented strain *Δbcelp4C* and the wild‐type strain. (Fig. 1A and B). A previous study had shown that sclerotial generation in dying host tissues plays an important role in the survival of *B. cinerea* (Willamson *et al*., 2007). We explored the function of *bcelp4* on sclerotial generation, the *bcelp4* deletion mutant was unable to produce any sclerotia, and in contrast, the wild‐type and the complemented strain *Δbcelp4C* produced vast sclerotia after incubation on PDA, MM and CM, respectively, in dark for 4 weeks (Fig. 1C and D). These results shown that *bcelp4* was required for vegetative growth and sclerotial formation in *B. cinerea*.

### Sensitivity of the *bcelp4* deletion mutant to various environmental stresses

**Figure 2:**
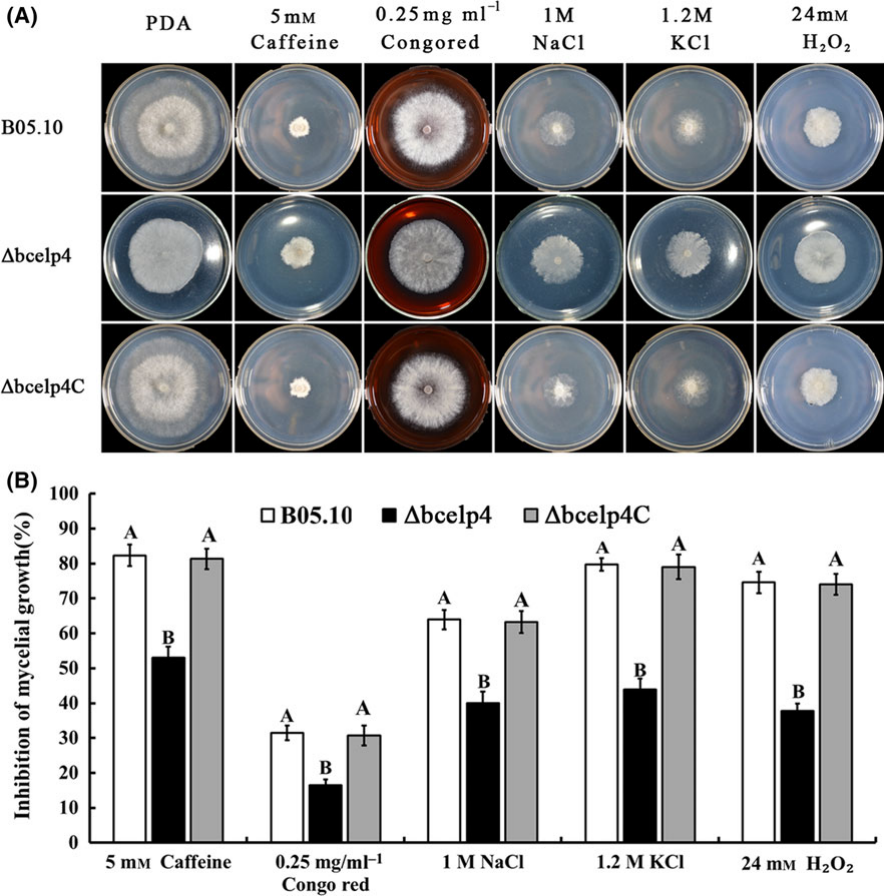
Sensitivity of B05.10, *Δbcelp4* and *Δbcelp4C* to various stress. A. Comparisons were performed on PDA medium modified with Congo red, caffeine, NaCl, KCl, paraquat and H_2_O_2_ at the content indicated in the figure. B. Inhibition of mycelial growth was analysed after each strain was incubated for 3 days on PDA supplement with different compound as described in the figure. Bars denote standard deviation from three experiments. Values on the bars followed by the same letter are not significantly different at *P* = 0.05. The comparison was performed between bar groups.

**Figure 3:**
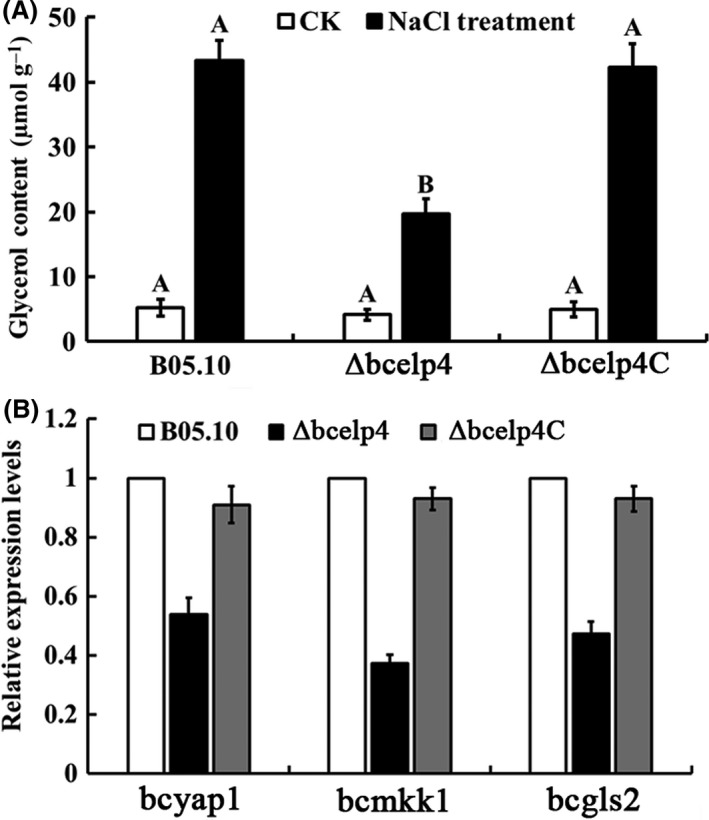
Involvement of *bcelp4* in glycerol accumulation and expression levels of various stresses response gene A. Glycerol content in mycelia of B05.10, *Δbcelp4* and *Δbcelp4C*. Bars denote standard errors from three repeated experiments. B. Expression levels of oxidative stress relation gene (*bcyap1*) and cell wall relation genes (*bcmkk1* and *bcgls2*) in each strain. RNA samples were isolated from mycelia treated with NaCl (1.0 M) and Congo red (0.25 mg ml^−1^) for 2 h respectively. Bars denote standard deviation from three repeated experiments. Values on the bars followed by the same letter are not significantly different at *P *= 0.05. The comparison was performed between values of the same treatment.

**Figure 4:**
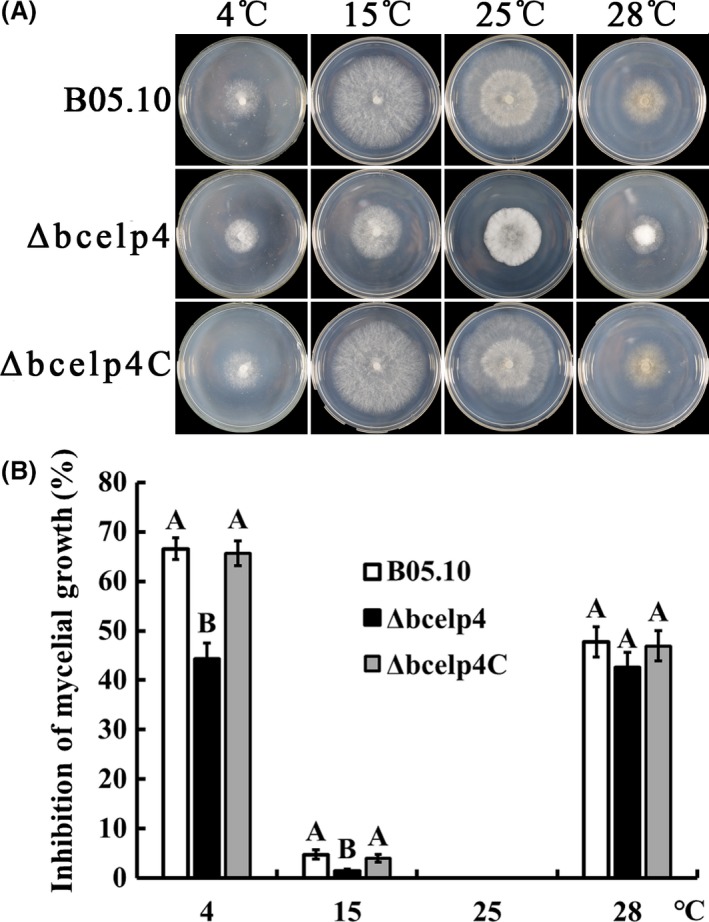
Sensitivity of B05.10, *Δbcelp4* and *Δbcelp4C* to thermal stress. A. Comparisons were made on PDA after each strain was incubated at 4, 15, 25 and 28°C for 3 days. B. Inhibition of mycelial growth compared with 25°C was measured after each strain was incubated for 3 days at 4, 15 and 28°C on PDA. Bars denote standard deviation from three experiments. Values on the bars followed by the same letter are not significantly different at *P* = 0.05. The comparison was performed between bar groups.

In a previous study, it was shown that the *elp4* plays an important role in the sensitivity of *S. cerevisiae* to various stresses (Krogan and Greenblatt, 2001). Therefore, we tested the sensitivity of the *bcelp4* deletion mutant to various environmental stresses including cell wall‐damaging agents (Caffeine and Congo red), osmotic and oxidative stresses. As shown in Fig. 2, the *Δbcelp4* revealed significantly decreased sensitivity to osmotic stresses generated by 1.0 M NaCl or 1.2 M KCl. It has been reported that osmotic stress can induce glycerol accumulation in fungi primarily triggered by activation of the HOG pathway (Fillinger *et al*., 2001; de Vries *et al*., 2003). Then, we tested the glycerol content in the *bcelp4* deletion mutant. As shown in Fig. 3A, in the absence of osmotic stress, very little glycerol was detected in the wild‐type stain or the *ΔBcElp4*. However, NaCl stress induced an eightfold increase in the glycerol content of the B05.10 strain but not to the same extent in the *bcelp4* deletion mutant. Additionally, as shown in Fig. 2, *Δbcelp4* also exhibited significantly decreased sensitivity to oxidative stress generated by 24 mM H_2_O_2_ or cell way‐damaging agent generated by 5 mM caffeine or 0.25 mg ml^−1^ Congo red. In a previous study, it was shown that *mkk1* and *gls2* were cell wall integrity core genes in *S. cerevisiae* (Rodriguez *et al*., 1985). In addition, the Yap1 protein was a relevant regulation factor in oxidative stress response in *S. cerevisiae* (Kuge *et al*., 1997; Lu *et al*., 2003; He and Fassler, 2005). To further investigate the role of *bcelp4* in the cell wall integrity and oxidative stress response in *B. cinerea*, we analysed the expression of *bcmkk1*,* bcgls2 and bcyap1*. Which were homologous to *mkk1*,* gls2* and *yap1* of *S. cerevisiae*, respectively. As shown in Fig. 3B, the expression levels of these three genes were down‐regulated in *Δbcelp4* relative to the wild‐type strain (ABI 7500 SDS software; Applied Biosystems, Foster City, CA, USA). Meanwhile, as shown in Fig. 4, *Δbcelp4* exhibited decreased sensitivity to low temperature (4°C and 15°C). The results indicated that the *bcelp4* gene product affects on the sensitivity of *B. cinerea* to various stresses.

### Effect of *bcelp4* deletion on aerial mycelia development of *B. cinerea*

**Figure 5:**
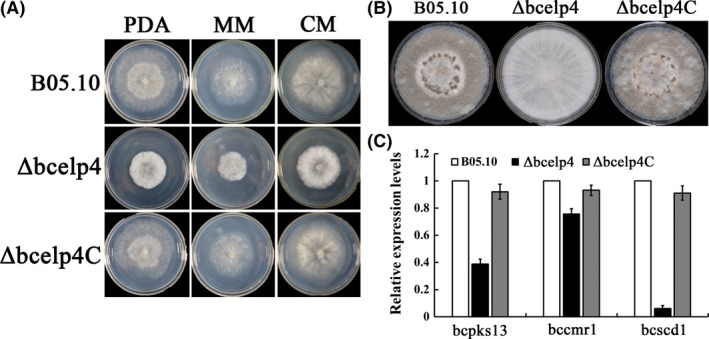
Effect of *bcelp4* on mycelial growth and hyphal melanization. A. B05.10, *Δbcelp4* and *Δbcelp4C* were grown on PDA, MM or CM at 25°C for 3 days. B. Comparisons of mycelial melanization among the wild‐type strain B05.10, the mutants *Δbcelp4* and the complemented strains *Δbcelp4C* after 10 days of incubation on PDA plates. C. Relative expression levels of three melanin biosynthesis‐related genes: *BcPks13*,* BcCmr1* and *BcScd1* in B05.10, *Δbcelp4* and *Δbcelp4C*. Bars denote standard deviation from three experiments.

Each strain was cultivated on PDA, MM and CM medium at 25°C in dark for 3 days. As shown in Fig. 5A, the *bcelp4* deletion mutant generated more white aerial mycelia than the wild‐type strain or the complemented strain. This result indicated that *bcelp4* is involved in mycelia differentiation in *B. cinerea*.

### Involvement of *bcelp4* in the regulation of mycelial melanization

To investigate the *bcelp4* involvement in the synthesis of melanin in mycelia, each of the strains were incubated on PDA at 25 °C for 10 days (Thompson *et al*., 2000). As shown in Fig. 5B, compared to the wild‐type strain B05.10 and the complemented strain *Δbcelp4C*, hyphal melanization of the *bcelp4* deletion mutant decreased when incubated in PDA medium. This phenotype was further confirmed by measuring the level of expression of the melanin biosynthetic related genes *bcpks13*,* BcCmr1* and *bcscd1* (Eliahu *et al*., 2007; Liu *et al*., 2011), which were lower in *Δbcelp4* than that in the wide‐type strain B05.10 (Fig. 5C). These results support that *bcelp4* is related to melanin synthesis in *B. cinerea*.

### Requirement of *bcelp4* in the virulence of *B. cinerea*

**Figure 6:**
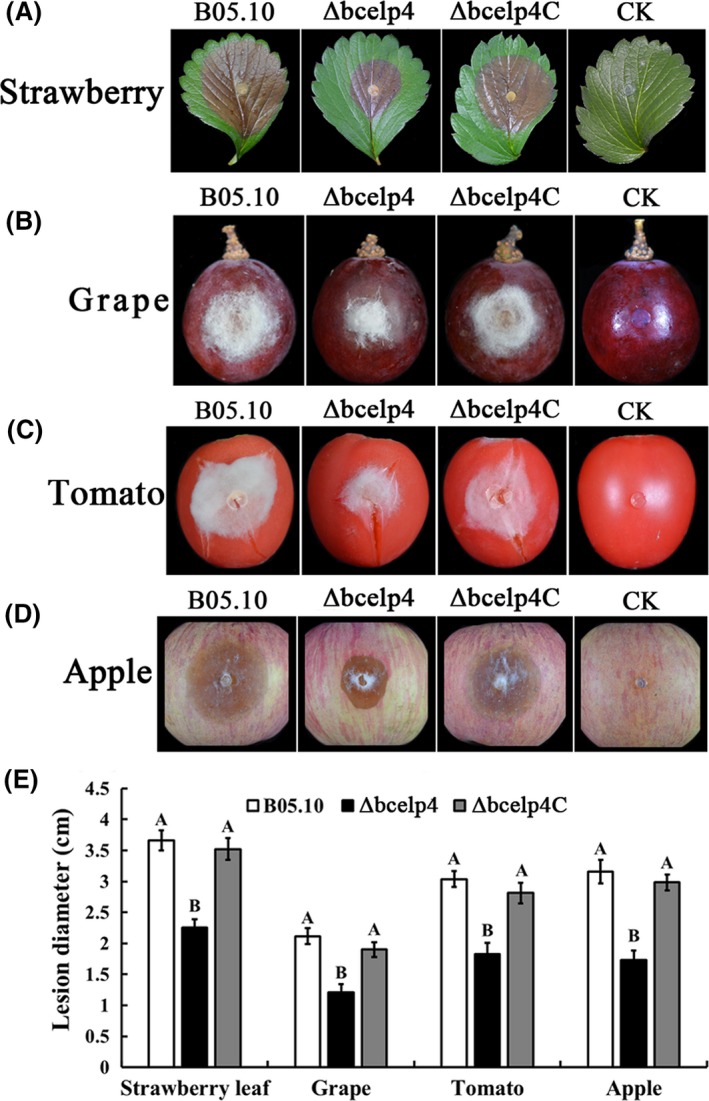
Virulence analysis on various plant tissue, following inoculation with the parental strain B05.10, *Δbcelp4* and *Δbcelp4C*. Agar plugs were used as negative controls (CK). A. Disease symptoms on wounded strawberry leaf after 80‐h inoculation (h.p.i.), B. wounded grape fruits 72 h.p.i, C. wounded tomato fruits wounded 72 h.p.i, D. wounded apple fruits 80 h.p.i. E. Diameter of disease lesion on various plant tissues. Bars denote the standard deviation of four repeated experiments. Values on the bars followed by the same letter are not significantly different at *P* = 0.05. The comparison was performed between bar groups.

The involvement of *bcelp4* in virulence was analysed on various host plant tissues. Mycelial plugs of each strain were inoculated on host plant tissues. On wounded grapes, apples, tomato and leaves of strawberry, the *bcelp4* deletion mutant caused significantly smaller disease lesions than the wild‐type strain B05.10 and complemented strain *Δbcelp4C* (Fig. 6) after inoculated for 72 h with 16 h of daylight. The results indicated that the *bcelp4* plays an important role in the virulence in *B. cinerea*.

## Discussion

A previous study had shown that Elongator is a histone acetyltransferase complex involved in elongation of RNA polymerase II transcription (Otero *et al*., 1999). In yeast, the deletion of any of the six Elongator protein subunit (*Elp1‐Elp6*) gene causes similar pleiotropic effects (Huang *et al*., 2005). In this study, we identified a *S. cerevisiae Elp4* homologous gene *bcelp4* in *B. cinerea*. The function analysis of *bcelp4* indicated that *bcelp4* is involved in various phenotypes in *B. cinerea*. A previous study showed that *elp4* affected vegetative growth in *S. cerevisiae* (Krogan and Greenblatt, 2001). Consistent with this observation is that the *bcelp4 B. cinerea* mutant showed significant defect on mycelia growth on PDA, MM or CM medium compared with the wild‐type strain B05.10 and the complemented strain *Δbcelp4C*. These results indicated that *elp4* played a relevant role in development in different fungi. In a previous study, it was shown that sclerotia play an important role in sexual reproduction during the development of apothecia in *B. cinerea* (Willamson *et al*., 2007). In this study, we found that the wild‐type strain and the complemented strain produced vast sclerotia, while the *Δbcelp4* mutant lost the ability of sclerotia formation. This result indicated that the *bcelp4* played an important role in sexual reproduction mechanism in *B. cinerea*.

In a previous study with *S. . cerevisiae* it was shown that the *elp4* was involved in sensitivity to various environmental stresses (Krogan and Greenblatt, 2001). In our study, the results support that the *bcelp4* deletion mutant showed significantly decreased sensitivity to cell wall‐damaging compounds such as Congo red or caffeine, oxidative stress generated by H_2_O_2_, osmotic stresses generated by NaCl or KCl and low temperature stresses generated by 4 or 15°C. Gene expression of *bcmkk1*,* bcgls2* and *bcyap1* was significantly decreased in *Δbcelp4* compared with that in B05.10 and *Δbcelp4C*. Additionally, when all of strains responded to osmotic stress, glycerol accumulation in the *bcelp4* deletion mutant was lower than that in wild‐type strain and the complemented strain. Based on these results, we deduced that the deletion of *bcelp4* may interfere with regulated factors in various stress responses in *B. cinerea* leading in the deletion mutant to decreased sensitivity to various stresses. This hypothesis needs to be further explored. In addition, we found that the *bcelp4* deletion mutant generated more aerial mycelia than that of wild‐type strain and the complemented strain. This result indicated that the *bcelp4* affects on the differentiation of mycelia vegetative growth in *B. cinerea*.

In the current study, we found that the *bcelp4* deletion mutant generated lower levels of melanin than wild‐type strain and the complemented strain. In many fungal species, *Paracoccidiodes brasiliensis*,* Cochliobolus heterostrophus* and *Cryptococcus gattii*, melanin has been shown to play multiple roles in providing defence against various environmental stresses such as oxidizing factors, UV light and ionizing radiation and it contributed to the ability of fungi to survive in harsh environments (Eisenman and Casadevall, 2012). As the *bcelp4* deletion mutant generated lower melanin than that of wild‐type strain and the complemented strain, we explored whether decreased melanin generation in the *bcelp4* deletion mutant conferred reduced sensitivity of *B. cinerea* to various environmental stresses, which was confirmed.

In current study, phenotypic characterization of *B. cinerea bcelp4* mutant showed that it was also involved in virulence. The reduced virulence of the *bcelp4* deletion mutant could be due to defects in multiple regulatory factors. First, the *bcelp4* deletion mutant grew significantly slower than the wild‐type strain or the complemented strain. We found also that the *Δbcelp4* showed decreased sensitivity to various environmental stresses, which play important roles in the interaction of fungal with host plants (Rolke *et al*., 2004; Motoyama *et al*., 2005; Arbelet *et al*., 2010), what might be related to the low virulence of this mutant on some plant tissues (Esberg *et al*., 2006).

In conclusion, we have compared *B. cinerea Δbcelp4* mutant with the wild‐type strain and have shown that *bcelp4* plays an important role in growth, melanin production, asexual development, responses to various stresses and virulence. Our results provided the basis for further exploration of function of Elongator complex in *B. cinerea* and may enhance the development of fungicides that target *bcelp4* for the control of plant diseases caused by *B. cinerea*.

## Experimental procedures

### Strains and culture conditions

*Botrytis cinerea* strain B05.10 isolated from grape in Germany was used as a wild‐type strain for the transformation experiments. The strain of this study was grown on potato dextrose agar (PDA) (200 g potato, 20 g glucose, 20 g agar and 1 l water), minimal medium (MM) (10 mM K_2_HPO_4_, 10 mM KH_2_PO_4_, 4 mM (NH4)_2_SO_4_, 2.5 mM NaCl, 2 mM MgSO_4_, 0.45 mM CaCl_2_, 9 μM FeSO_4_, 10 mM glucose and 1 l water, pH 6.9) or complete medium (CM) (1% glucose, 0.2% peptone, 0.1% yeast extract, 0.1% casamino acids, nitrate salts, trace elements, 0.01% vitamins and 1 l water, pH 6.5) for mycelial growth assay. Additionally, each stain was grown on sterilized potato fragments and PDA for conidiation tests.

Mycelial growth assays under different condition were performed on PDA plates modified with the following: caffeine, Congo red, H_2_O_2_, NaCl and KCl at the concentrations indicated in the figure legends. Each plate was inoculated with a 5‐mm‐diameter mycelial plug taken from the edge of a 3‐day‐old colony grown on PDA. The percentage of inhibition of mycelial radial growth (PIMG) was calculated using the formula, PIMG = [(C – N)/(C – 5)] × 100, where C is the colony diameter of the untreated control and N is that of a treatment. Each experiment was repeated three times independently.

### Deletion of the *bcelp4* gene and complemented mutant

To investigate the functions of *bcelp4* in *B. cinerea*, we constructed a *bcelp4* deletion mutant. The gene replacement cassette was generated as described previously (Laleve *et al*., 2014; Zheng *et al*., 2014). First, 1409‐bp upstream and 1362‐bp downstream flanking fragment of *bcElp4* were amplified from the wild‐type strain B05.10 genomic DNA with the primer pairs P3/P4 and P5/P6 respectively (Fig. S2, Table S1). Subsequently, the 1764‐bp *HPH* cassette (resistance to hygromycin B) containing a *trpC* promoter was amplified form the pKHT plasmid with the primer pair P7/P8 according to previous study (Duan *et al*., 2013). Then, the two *bcelp4* flanking fragments were mixed with the *HPH* cassette in a molar proportion of 1:1:3 and used as a template for the fusion PCR (Fig. S2) (Yu *et al*., 2004). The 4676‐bp DNA fragment amplified from the fusion PCR production using the primer pair P9/P10 and used for transforming protoplasts of the wild‐type strain B05.10.

Protoplast generation and transformation of *B. cinerea* were performed referring to previous study with some modification (Schulze *et al*., 2001; Wang *et al*., 2015). Mycelial plugs cut from the edge of 3‐day‐old colony on PDA were placed in 250‐ml flask containing 100 ml of liquid YEPD (10 mg/ml peptone, 3 mg/ml yeast extract, 20 mg/ml glucose). After the flasks have been shaken at 175 rpm and 25°C for 18 h, fresh mycelia were collected using a sterile filter and washed twice with distilled water. Then, 0.15 g of fresh mycelia was incubated with 15 ml 2% lysing enzymes buffer (0.6 M KCl, 50 mM CaCl_2_; Sigma, St Louis, MO, USA). After 2 h at 30°C and 85 rpm, the enzyme solution was filtered to eliminate mycelial residues. The protoplasts in the filtrate were washed twice with STC buffer (0.8 M sorbitol, 0.05 M Tris, pH 8.0, 50 mM CaCl_2_) and resuspended in (STC with 40%, w/v, PEG6000) buffer (STC: SPTC = 4:1). The transformation of protoplasts was performed as described previously (Zheng *et al*., 2014). For transformation, 10^7^ in 500 μl of SPTC buffer and 35 μg replacement vector in 20 μl of spermidine were mixed and incubated on ice for 90 min; 1 ml SPTC was added into the suspension and incubated at 25°C for 30 min. Protoplasts were mixed into 200 ml RM medium (0.5 g/l yeast extract, 0.5 g/l casein hydrolysate, 0.7 M sucrose and 16 g/l agar powder) at 42°C, plated on petri plate (15 ml per plate) and incubated at 25°C for 16 h, and then, RM plates were overlaid with 10 ml of SRM medium (0.5 g/l yeast extract, 0.5 g/l casein hydrolysate, 1 M sucrose and 12 g/l agarose) containing 100 μg/ml hygromycin B. After incubation at 25°C for 4 days, the transformants were transferred to fresh PDA containing 100 μg/ml hygromycin B for PCR detection assay. Transformants indicating the homologous replacement of the vector were detected by PCR amplification of genomic DNA using different special primer pairs, P11/P12, P13/P14, P15/P16 and P17/P18 (Table S1, Fig. 2A) and confirmed by southern analysis of genomic DNA digested with EcoRV and hybridized with a labelled probe generated by PCR amplification using primer pair P21/P22 (Table 1, Fig. 1A).

To confirm that the phenotype of the *bcelp4* deletion mutant resulted from deletion of the gene, the *bcElp4* deletion mutant was complemented with the full‐length *bcElp4* gene refer to a previous study (Duan *et al*., 2013). The *bcelp4* complement plasmid pNEO‐*bcelp4*‐Com was constructed using the backbone of pCAMBIA 1300 (CAMBIA, Canberra, Australia). First, a *BstXI*‐*XhoI* NEO cassette containing a trpC promoter (resistance to neomycin) was amplified form plasmid PII99‐Pro (DOHH) GFP with primers P19/P20 (Table S1) and cloned into the *BstXI*‐*XhoI* site of pCAMBIA 1300 to create plasmid pNEO. Then, the full‐length *bcelp4* gene including the 415‐bp upstream and 319‐bp terminator regions was amplified from genomic DNA of the wild‐type strain with primers P19/P20 (Table S1) and subsequently cloned into the *Sma I* – *Hind III* site of pNEO to generate the complement plasmid pNEO‐*bcelp4*‐Com. Before the plasmid, pNEO‐*bcelp4*‐Com was transformed into strain *Δbcelp4*, and *bcelp4* in this plasmid was sequenced to ensure sequence correctness. Transformation of *Δbcelp4* with plasmid pNEO‐*bcelp4*‐Com was conducted as described above, except that neomycin (100 μg/ml) was used as a selection agent.

### Nucleic acid manipulations

Fungal genomic DNA was extracted according to the previous described method (McDonald and Martinez, 1990). Southern blot hybridization analysis of *bcelp4* gene in the transformants of *B. cinerea* was performed using a 706‐bp fragment downstream of *bcelp4* as a probe. The probe was labelled with digoxigenin using the High Prime DNA labelling and detection starter kit II referring to the protocol of the manufacturer (Roche Diagnostics, Mannheim, Germany). DNA isolated from *B. cinerea* was digested with *EcoR V* and used for southern hybridization analysis.

### Quantitative RT‐PCR assay

To isolate total RNA, the mycelial plugs of each strain were inoculated into liquid YEPD (10 mg/ml peptone, 3 mg/ml yeast extract, 20 mg/ml glucose) and cultured at 25°C for 2 days in a shaker (175 rpm). RNA was isolated from mycelia with the RNeasy kit (Tiangen Biotech. Co., Beijing, China). First‐strand cDNA was synthesized with the PrimeScript^®^ RT reagent kit (TaKaRa). The real‐time PCR amplifications were conducted in an ABI 7500 detection system (Applied Biosystems) (Zheng *et al*., 2014). The primers used for Quantitative RT‐PCR assay are showed in Table S1. The expression level of the measured gene in all samples was normalized to *actin* gene expression, and relative changes in gene expression levels were analysed by ABI 7500 SDS software (Applied Biosystems), which automatically generates the baseline. Data from three biological replicates were applied to calculate the mean standard deviation.

### Assay of intracellular glycerol accumulation

To determinate glycerol accumulation, each strain was cultivated in YEPD (10 mg/ml peptone, 3 mg/ml yeast extract, 20 mg/ml glucose) for 2 days at 25°C in a shaker. After treatment with 1.2 M NaCl, mycelia of each strain were harvested and ground in liquid nitrogen. The mycelial powder (0.15 g) was transferred to a 2‐ml microcentrifuge tube containing 0.5 ml of glycerol isolation buffer (Applygen, Beijing, China). After vortexing three times for 35 s each, the tubes were centrifuged at 6000 ***g*** for 25 min. The supernatant was transferred to a 1.5‐ml tube, and then, 10 μl of each supernatant in tube was mixed with 190 μl detection buffer from glycerol determination kit (Applygen). After the mixture was incubated for 15 min at 37°C, the glycerol concentration was determined by a microplate reader (SpectraMax M5) at 550 nm (Yang *et al*., 2012). The experiment was independently repeated twice.

### Virulence assays

To investigate the role of *bcelp4* in the virulence of *B. cinerea*, 3‐week‐old strawberry leaves, and grape, tomato and apple fruits were inoculated with 5‐mm‐diameter mycelial plugs cut from 3‐day‐old colony margin, respectively. Before inoculation, the leaves and fruits were wounded with a sterile needle tip to facilitate the penetration of the fungus into host plant tissue. Inoculated tissues were cultivated at 25°C with 16 h of daylight (Yang *et al*., 2012). Diameter of disease lesions was recoded after the indicated times in figure legends. The experiment was repeated three times.

## Conflict of Interest

None declared.

## Supporting information


**Fig. S1.** The structure of *bcelp4* and amino acid alignments of *Botrytis cinerea elp4* (*bcelp4*) with those of *Neurospora crassa elp4* (*ncelp4*), *Magnaporthe oryzae elp4* (*moelp4*), *Fusarium graminearum elp4* (*fgelp4*), *Saccharomyces cerevisiae elp4* (*scelp4*) and *Sclerotinia sclerotiorum elp4* (*sselp4*). Boxshade program was used to highlight identical (black shading) and similar (grey shading) amino acids.

**Fig. S2.** Generation and identification of the *bcelp4* deletion mutant of *Botrytis cinerea*.

**Fig. S3.** Identification of the *bcelp4* deletion mutant of *Botrytis cinerea* with PCR method.

**Table S1.** Primers used in this study.
