# Supplementary figures and images for: Involvement of BcElp4 in vegetative development, various environmental stress response and virulence of Botrytis cinerea
Source: Microb Biotechnol. 2017 May 5;10(4):886–95. doi: 10.1111/1751-7915.12720 (PMC5481526; doi:10.1111/1751-7915.12720)

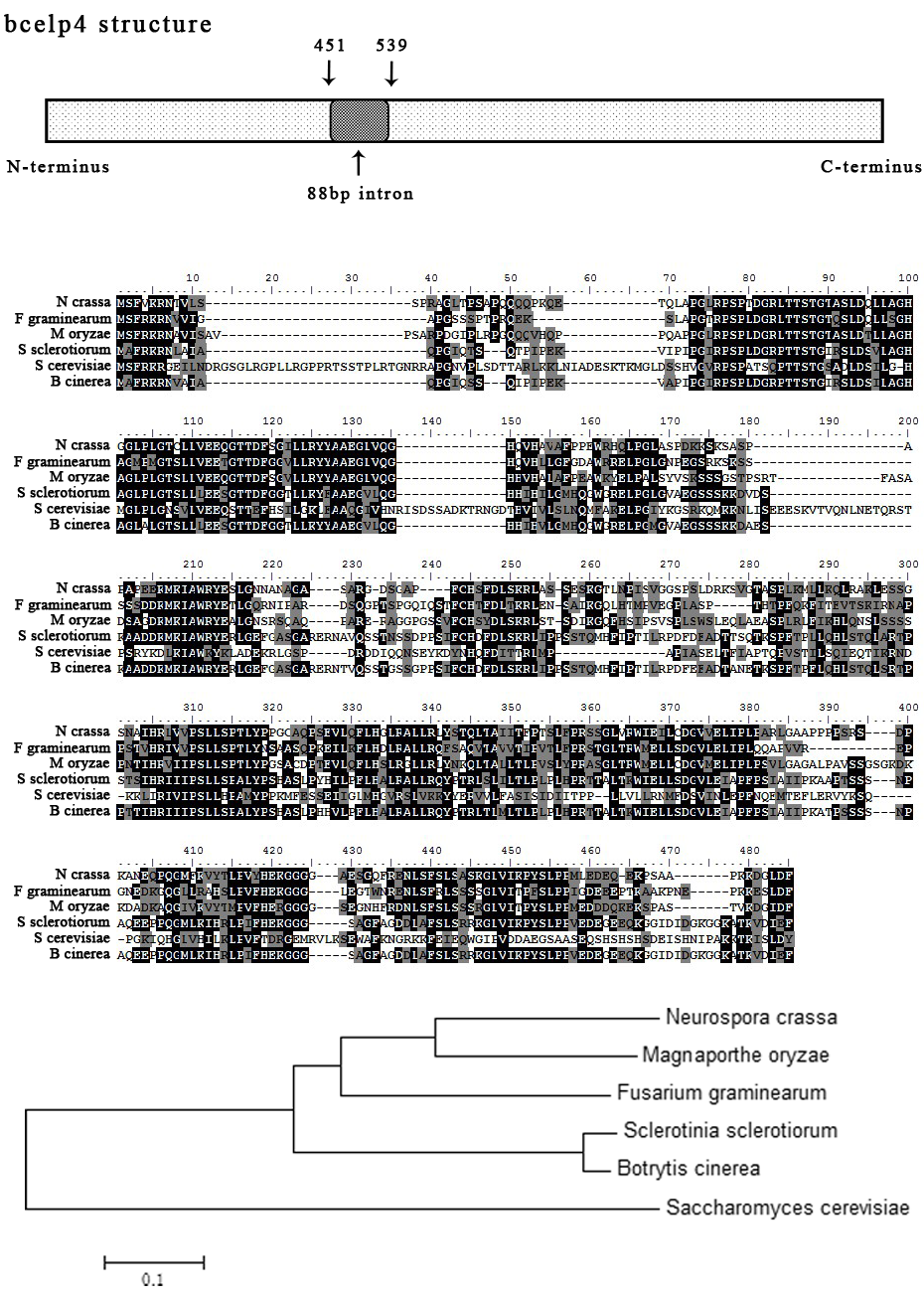

Supplement: Supplementary file 1 — Fig. S1. The structure of bcelp4 and amino acid alignments of Botrytis cinerea elp4 (bcelp4) with those of Neurospora crassa elp4 (ncelp4), Magnaporthe oryzae elp4 (moelp4), Fusarium graminearum elp4 (fgelp4), Saccharomyces cerevisiae elp4 (scelp4) and Sclerotinia sclerotiorum elp4 (sselp4). Boxshade program was used to highlight identical (black shading) and similar (grey shading) amino acids. [file MBT2-10-886-s001.tif]

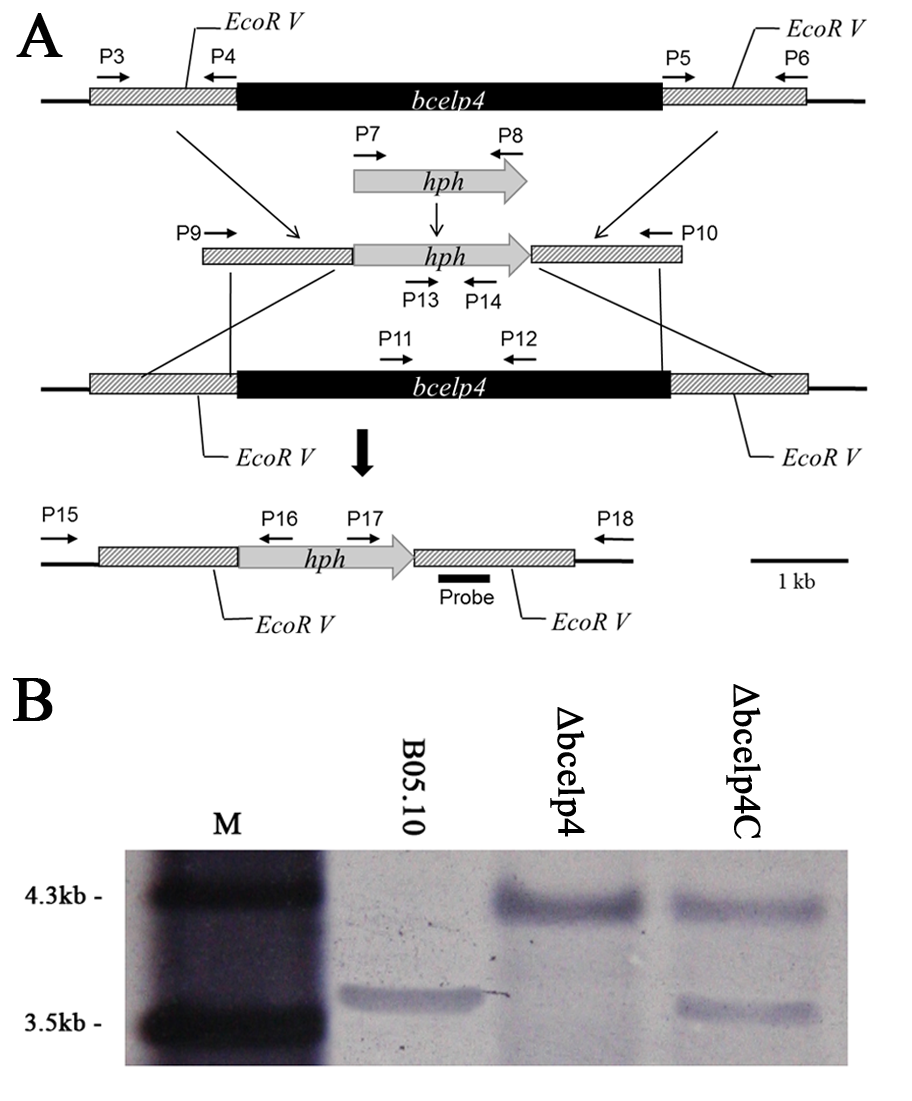

Supplement: Supplementary file 2 — Fig. S2. Generation and identification of the bcelp4 deletion mutant of Botrytis cinerea. [file MBT2-10-886-s002.tif]

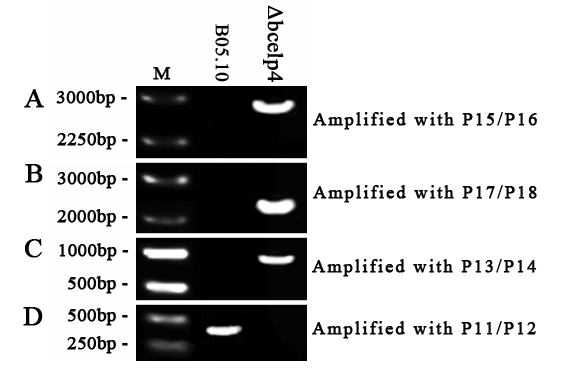

Supplement: Supplementary file 3 — Fig. S3. Identification of the bcelp4 deletion mutant of Botrytis cinerea with PCR method. [file MBT2-10-886-s003.tif]
