# Supplementary material for: Involvement of BcElp4 in vegetative development, various environmental stress response and virulence of Botrytis cinerea
Source: Microb Biotechnol. 2017 May 5;10(4):886–95. doi: 10.1111/1751-7915.12720 (PMC5481526; doi:10.1111/1751-7915.12720)
Supplement: Supplementary file 4 — Table S1. Primers used in this study. [file MBT2-10-886-s004.docx]

**Table S1**

| Primer code Sequence (5′→3′) | Relevant characteristic |
| --- | --- |
| P1 ATGGCTTTTCGAAAACGCAACG | Amplify the full sequence of cDNA and genomic DNA the *bcelp4* gene |
| P2 CTAAAATTCTATGTCGACTTTGG |  |
| P3TTGGAGTTGCCTGCTGAT | Amplify the left homologous arm of the *bcelp4* gene of *B. cinerea* (1409 bp) |
| P4CCACCAGCCAGCCAACAGCTCCCAGGTCTTTCTTTTATTTTATGGG |  |
| P5 CAATACGCAAACCGCCTCTCCCCAGGAAACTTTACTCGGAATC | Amplify the right homologous arm of the *bcelp4* gene of *B. cinerea* (1362 bp) |
| P6 CACTTCGGCAACACTACC |  |
| P7 GGGAGCTGTTGGCTGGCTGGTGG | Amplify the *hph* gene (1764 bp) |
| P8 GGGGAGAGGCGGTTTGCGTATTG |  |
| P9 GCCTGCTGATGTCATTCC | Amplify the knockout vector of the  *bcelp4* gene of *B. cinerea* (4476 bp) |
| P10 ACTTCGGCAACACTACC |  |
| P 11 GTGGTGCTAGAGGTGGGT | Amplify a partial fragment of the *bcelp4* gene of *B. cinerea* (345 bp) |
| P 12 GATGCGGTGGATAGTTGTA |  |
| P 13 actctattcctttgccctcgg | Amplify a partial fragment of the *hph* gene (981 bp) |
| P 14 GAAAAGTTCGACAGCGTCTCC |  |
| P 15 TGTTCGGCATTCTGGGTT | Confirm whether the *hph* genes homologously replaced the *bcelp4* gene of *B. cinerea* (2688bp) |
| P 16 CTGGCAAACTGTGATGGAC |  |
| P 17 gtgccgataaacataa | Confirm whether the *hph* genes homologously replaced the  *bcelp4* gene of *B. cinerea* (2399bp) |
| P 18 ACATCTCAAGCCTCAT |  |
| P19 TCCcccgggACCGTTGGCGGCTTGTATT | Amplify the *bcelp4* gene (include the control region of the *BcElp4* gene) (1974 bp) |
| P20 CCCaagcttCTCCATCCCTCGCACATCA |  |
| P21 GATGGAGATGGTTCGGGATA | Amplify a probe for Southern blotting (706 bp) |
| P22 GGGATGATAGGTTGTGGAGC |  |
| P25 TAGGTGATTTGGGACAACAGAG | Amplify the *bcyap1* gene for quantitative real-time PCR |
| P26 GTCTCTCAATGGTGCGGATAG |  |
| P27 GCTACGCCTATGGGAAGTAATG | Amplify the *bcmkk1* gene for quantitative real-time PCR |
| P28 CTACTTTCGCTTCCTCCACTTG |  |
| P29 GGCAAGTTGAAGGAGCAAATC | Amplify the *bcgls* gene for quantitative real-time PCR |
| P30 ATCTGGTGCGTGTGTGATAG |  |
| P31 TCACGCATTGACACCTACACA | Amplify the *bccmr1*  gene for quantitative real-time PCR |
| P32 TTCCATGTTCCAATTCCTCG |  |
| P33 TCAACCACACGGACCATATC | Amplify the *bcpsk13*  gene for quantitative real-time PCR |
| P34 CGCCATCACCAAGAAGACC |  |
| P35 TGCCCGCTTCCCAATTCAT | Amplify the *bcscd1*  gene for quantitative real-time PCR |
| P36 TCCATCACCCTCATTCCATCT |  |
| P37 CGTCTGGATTGGTGGTTCTATT | Amplify the reference gene actin for quantitative real-time PCR |
| P38 ACTCGTCGTACTCTTGCTTTG |  |
